# Predictors of Bone Metastases at ^68^Ga-PSMA-11 PET/CT in Hormone-Sensitive Prostate Cancer (HSPC) Patients with Early Biochemical Recurrence or Persistence

**DOI:** 10.3390/diagnostics12061309

**Published:** 2022-05-24

**Authors:** Guido Rovera, Serena Grimaldi, Sara Dall’Armellina, Roberto Passera, Marco Oderda, Giuseppe Carlo Iorio, Alessia Guarneri, Paolo Gontero, Umberto Ricardi, Désirée Deandreis

**Affiliations:** 1 Nuclear Medicine, Department of Medical Sciences, AOU Città Della Salute e Della Scienza di Torino, University of Turin, 10126 Turin, Italy; guido.rovera@unito.it (G.R.); sgrimaldi@cittadellasalute.to.it (S.G.); sara.dallarmellina@unito.it (S.D.); desiree.deandreis@unito.it (D.D.); 2 Urology, Department of Surgical Sciences, AOU Città Della Salute e Della Scienza di Torino, University of Turin, 10126 Turin, Italy; marco.oderda@unito.it (M.O.); paolo.gontero@unito.it (P.G.); 3 Department of Oncology, University of Torino, 10125 Torino, Italy; giuseppecarlo.iorio@libero.it (G.C.I.); alessia.guarneri.ag@gmail.com (A.G.); umberto.ricardi@unito.it (U.R.)

**Keywords:** prostatic neoplasm, hormone-sensitive prostate cancer, PSMA PET, bone disease, predictive model

## Abstract

Prostate-specific-membrane-antigen/positron-emission-tomography (PSMA-PET) can accurately detect disease localizations in prostate cancer (PCa) patients with early biochemical recurrence/persistence (BCR/BCP), allowing for more personalized image-guided treatments in oligometastatic patients with major impact in the case of bone metastases (BM). Therefore, this study aimed to identify predictors of BM at PSMA-PET in early-BCR/BCP hormone-sensitive PCa (HSPC) patients, previously treated with radical intent (radiotherapy or radical prostatectomy ± salvage-radiotherapy (SRT)). A retrospective analysis was performed on 443 ^68^Ga-PSMA-11-PET/CT scans. The cohort median PSA at PET-scan was 0.60 (IQR: 0.38–1.04) ng/mL. PSMA-PET detection rate was 42.0% (186/443), and distant lesions (M1a/b/c) were found in 17.6% (78/443) of cases. BM (M1b) were present in 9.9% (44/443) of cases, with 70.5% (31/44) showing oligometastatic spread (≤3 PSMA-positive lesions). In the multivariate binary logistic regression model (accuracy: 71.2%, Nagelkerke-R^2^: 13%), T stage ≥ 3a (OR: 2.52; 95% CI: 1.13–5.60; *p* = 0.024), clinical setting (previous SRT vs. first-time BCR OR: 2.90; 95% CI: 1.32–6.35; *p* = 0.008), and PSAdt (OR: 0.93; 95% CI: 0.88–0.99; *p* = 0.026) were proven to be significant predictors of bone metastases, with a 7% risk increment for each single-unit decrement of PSAdt. These predictors could be used to further refine the indication for PSMA-PET in early BCR/BCP HSPC patients, leading to higher detection rates of bone disease and more personalized treatments.

## 1. Introduction

Accurate disease restaging represents a key step in the appropriate management of high-risk prostate cancer (PCa) patients with either biochemical recurrence (BCR) or persistence (BCP). Conventional imaging, such as computed tomography (CT) and bone scan, has shown suboptimal accuracy in disease localization compared to molecular imaging [1,2,3], and radiopharmaceuticals, such as ^11^C-choline and ^18^F-fluciclovine, could not reach high detection rates at low prostate-specific antigen (PSA) values (<2.0 ng/mL) [4,5].

In this context, molecular imaging with prostate-specific membrane antigen/positron emission tomography (PSMA-PET) has shown promising results for detecting loco-regional and distant metastases [6,7,8], and the European Association of Urology (EAU) Guidelines recommend performing PSMA-PET in patients with PSA failure after radical treatment, if the results could influence subsequent treatment decisions [9].

Thanks to a more accurate detection of disease localizations, molecular imaging with PSMA-PET can significantly influence clinical management [10,11,12], leading to more personalized therapies. Specifically, in patients with oligometastatic spread, PSMA-PET was successfully used to guide stereotactic ablative radiotherapy/stereotactic body radiation therapy (SABR/SBRT) [13,14,15,16]. In the ORIOLE phase II trial [16], SABR improved outcomes with significant advantages in terms of median progression-free survival (PFS) (unreached at 24 months follow-up vs. 11.8 months; HR 0.26; 95% CI: 0.09–0.76; *p* = 0.006) and distant metastasis-free survival (29.0 vs. 6.0 months; HR 0.19; 95% CI: 0.07–0.54; *p* < 0.001) in men who received consolidation of all of the disease localizations detected by PSMA-PET (baseline data blinded by protocol), supporting the use of molecular imaging in conjunction with metastasis-directed therapy (MDT) for patients with oligometastatic PCa. With regards to bone-only oligometastatic PCa patients, previous studies [14] also documented PSMA-PET guided SBRT to be an effective treatment, with a 2-year PFS rate of 72.0%, a PSA decline in 75.7% of patients, and a 2-year local control rate per lesion of 95.4%.

The identification of predictive factors, in particular of PCa bone involvement at molecular imaging, could help to refine the indication for PSMA-PET in early-BCR/BCP HSPC-patients, leading to higher detection rates, more personalized treatments, and higher cost-effectiveness from a patient and health-care perspective [17].

Therefore, this study was aimed to identify predictors of bone metastases at PSMA-PET in early-recurrent/persistent hormone-sensitive prostate cancer (HSPC) patients.

## 2. Materials and Methods

### 2.1. Study Design

A retrospective analysis was conducted on four hundred forty-three (443) consecutive ^68^Ga-PSMA-11-PET/CT scans performed between November 2016 and December 2021 at our institution (Department of Nuclear Medicine, University Hospital of Turin) in HSPC patients with early biochemical recurrence/persistence, according to the Guidelines.

Inclusion criteria were: (1) histologically proven PCa; (2) previous treatment with radical intent, either radical prostatectomy (RP) or radiotherapy (RT); (3) proven biochemical recurrence (BCR) or biochemical persistence (BCP), as defined by the EAU Guidelines [9]; (4) hormone-sensitive prostate cancer (HSPC), not treated with androgen deprivation therapy (ADT) during the 6 months preceding the PET scan; (5) PSA < 2.5 ng/mL or any PSA in case of negative choline-PET/CT or RT as primary therapy. Exclusion criteria were: (1) patients not eligible for salvage therapy; (2) inability to undergo a PET/CT scan; (3) castration resistant PCa (CRPC); (4) concurrent administration of androgen-receptor targeted therapy or chemotherapy. This retrospective analysis was conducted in conformance with the Helsinki Declaration and, according to Italian law (Italian Drugs Agency (AIFA)], Guidelines for Observational Studies, 20 March 2008), no formal IRB/IEC approval was needed.

### 2.2. Objectives

The primary objective of this study was to identify potential independent predictors of bone metastatic involvement in a cohort of hormone-sensitive prostate cancer (HSPC) patients undergoing ^68^Ga-PSMA-11 PET/CT for PSA failure after radical treatment.

### 2.3. Procedures and Image Interpretation

The ^68^Ga-PSMA-11 was synthesized in the radiochemistry laboratory of the Division of Nuclear Medicine of the AOU Città della Salute e della Scienza, University of Turin, as previously reported [11], in accordance with procedure guidelines [18,19]. All patients were injected intravenously with a 1.8–2.2 MBq/kg dose of ^68^Ga-PSMA-11 and received intravenous hydration with 0.5 L saline solution during uptake. Informed consent was obtained from all of the subjects before administration. No specific patient preparation was needed before the procedure, and no administration of furosemide or oral contrast media was required.

The ^68^Ga-PSMA-11 PET was performed in accordance with standard techniques, as previously reported [11], using dedicated tomographs (Gemini Dual and Vereos, Philips HealthCare, Cambridge, MA, USA). Attenuation correction of the PET emission data was performed by acquiring a low-dose CT scan. If standard images proved to be inconclusive, late pelvic scans were acquired at 120 (±15) minutes post-injection, 6 min per bed position, two beds centered on the pelvis.

Two experienced nuclear medicine physicians independently reviewed the PET/CT images, and any resulting discrepancy was solved by consensus. In accordance with the E-PSMA procedure Guidelines [19,20], a per-region analysis was performed, and prostate cancer lesions were suspected in the case of focal tracer uptake (higher than surrounding background) not corresponding to the physiological areas of radiotracer localization.

### 2.4. Statistical Analysis

For each patient, the collected data included information about disease staging, histopathologic grading, previous treatments, PSA kinetics, and PSMA-PET result.

Three different clinical settings of PSA failure were identified by the uro-oncological tumor board (genitourinary oncology group, AOU Città della Salute e della Scienza, University Hospital of Turin, Turin, Italy): first-time BCR (subgroup 1), defined as rising PSA levels ≥ 0.2 ng/mL in patients treated with RP or PSA levels ≥ 2 ng/mL above the nadir in case of primary RT; PSA recurrence after prostate-bed SRT (subgroup 2), defined as a PSA rise ≥ 0.2 ng/mL above the PSA nadir after SRT; BCP after RP (subgroup-3), defined as PSA ≥ 0.1 ng/mL, at least 6 weeks after RP.

At baseline, population characteristics were presented as absolute/relative frequencies for categorical variables and median (Inter Quartile Range (IQR)) for continuous ones. The PSA doubling time estimations were performed in accordance with Khan et al. [21], as previously documented [11].

Inferential statistics was performed using the Mann–Whitney test for continuous covariates, and the Fisher’s exact test for categorical ones, respectively.

The likelihood of bone disease at PSMA-PET was estimated by a complete series of uni- and multi-variate binary logistic regression models. While the dependent variable was the PSMA-PET bone metastatic status (M1b: positive vs. negative), the potential determinants were T stage (≥3a), ISUP grade (≥4), PSA doubling time (as a continuous covariate) and clinical setting (first-time BCR, previous salvage-radiotherapy [SRT], BCP). The number of potential determinants in the multivariate analysis was limited in order to preserve a 1: 10 ratio between predictors and events (M1b cases).

All reported *p*-values were two-sided, at the conventional 5% significance level. Data were analyzed as of January 2022 using IBM SPSS Statistics for Windows, version 26.0 (IBM Corp., Armonk, NY, USA).

## 3. Results

### 3.1. Cohort Characteristics

Four hundred forty-three (443) ^68^Ga-PSMA-11-PET/CT scans were performed in HSPC patients with early BCR/BCP and were considered eligible for the primary endpoint analysis. Table 1 shows the demographic and clinical characteristics of the study cohort. The median PSA was 0.60 (IQR: 0.38–1.04) ng/mL at the time of the PET scan, while the median PSAdt was 8.20 (IQR: 4.15–14.55)] months. Clinical settings of PSA relapse were distributed as follows: 215 first-time BCR, 174 BCR after salvage-radiotherapy (SRT), and 54 BCP cases.

### 3.2. PSMA-PET Results

**Figure 1:**
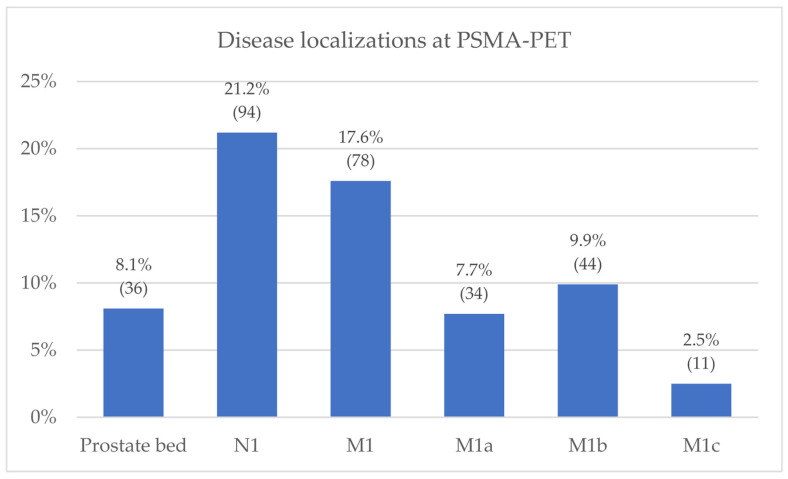
PSMA-PET results according to miTNM classification (*N* = 443).

**Figure 2:**
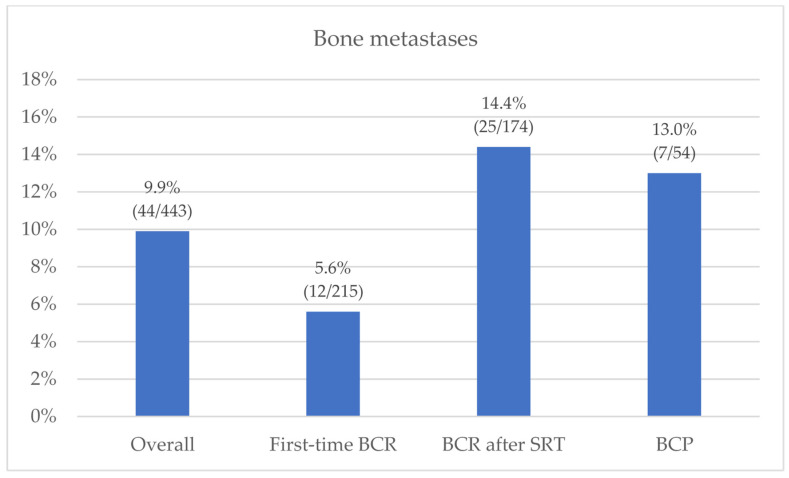
Bone metastases prevalence at PSMA-PET, stratified by clinical setting (*N* = 443).

The overall PSMA-PET detection rate was 42.0% (186/443). In accordance with molecular imaging TNM (miTNM) definition [19,20], the identified PCa lesions were categorized as follows: prostate bed (miTr) in 8.1% of cases (36/443); pelvic nodes (miN1) in 21.2% (94/443); extra-pelvic nodes (miM1a) in 7.7% (34/443); bone metastasis (miM1b) in 9.9% (44/443); and visceral non-nodal metastasis (miM1c) in 2.5% (11/443). Overall, 17.6% (78/443) of cases showed disease involvement outside the pelvis (miM1a, miM1b, miM1c), mostly oligometastatic (≤3 PSMA-positive localizations—80.8% (63/78)) (Figure 1). The prevalence of bone metastases at PET imaging varied among different clinical settings: 5.6% (12/215) in subgroup 1 (first-time BCR); 14.4% (25/174) in subgroup 2 (BCR after SRT); and 13.0% (7/54) in subgroup 3 (BCP), as detailed in Figure 2. Overall, 73% (32/44) of the miM1b cases showed exclusive skeletal involvement, and oligometastatic spread was found in 70.5% (31/44) of miM1b cases.

### 3.3. Predictors Analysis

The Mann–Whitney/Fisher’s exact test proved the T stage ≥ 3a (*p* = 0.009), ISUP-grade ≥ 4 (*p* = 0.013), clinical-setting (first-time BCR vs. previous SRT vs. BCP, *p* = 0.010), PSA doubling-time (PSAdt, *p* < 0.001), and PSA value at PET-scan (*p* = 0.015) to be significantly associated with bone disease at PSMA-PET. On the contrary, no significant associations were observed when stratifying the population by N-stage at diagnosis or primary treatment (radiotherapy/radical prostatectomy ± PLND).

In the univariate binary logistic regression model series, T stage ≥ 3a (OR 2.59; 95% CI: 1.26–5.30; *p* = 0.009), ISUP grade ≥ 4 (OR 2.37; 95% CI: 1.24–4.54; *p* = 0.009), clinical setting (previous SRT vs. first-time BCR OR 2.83; 95% CI: 1.38–5.83; *p* = 0.005, BCP vs. first-time BCR OR 2.52; 95% CI: 0.94–6.74; *p* = 0.066), and PSAdt (OR 0.91; 95% CI: 0.86–0.97; *p* = 0.004) proved to be significant predictors of bone metastases.

In the multivariate model (accuracy: 71.2%, Nagelkerke R^2^: 13%, sensitivity: 68.4%, specificity: 71.6%) significant results were confirmed for the T-stage (OR: 2.52; 95% CI: 1.13–5.60; *p* = 0.024), PSAdt (OR: 0.93; 95% CI: 0.88–0.99; *p* = 0.026), and clinical setting (previous SRT vs. first-time BCR OR: 2.90; 95% CI: 1.32–6.35; *p* = 0.008), but not for ISUP-grade (*p* = 0.146).

Based on this predictive model, a PSAdt decrease of one month would result in a 7% increment in the likelihood of bone metastatic involvement at PSMA-PET. These results are reported in detail in Table 2.

## 4. Discussion

This study represents a retrospective analysis of four hundred forty-three (443) ^68^Ga-PSMA-11-PET/CT scans performed in BCR/BCP HSPC patients previously treated with radical intent (RT or RP ± SRT). Our study showed a PSMA-PET overall detection rate of 42.0% (186/443). Although higher detection rates were previously reported in literature [22], our study investigated a cohort composed exclusively by HSPC ADT-free patients at an early stage of recurrence and eligible for salvage therapy. Thus, a higher proportion of negative scans is expected, due to lower PSA levels, lower tumor burdens, and a higher chance of micro-metastatic disease. Considering the previous findings in cohorts with comparable characteristics [5,7,23], the PSMA-PET positivity rate reported in our study falls in line with previous literature data.

PSMA-PET showed systemic disease recurrence (M1a/b/c) in 17.6% (78/443) of the study cohort, while skeletal involvement was detected in 9.9% (44/443) of the cases, mostly oligometastatic. These data are in accordance with a previous study from Calais et al. [5], conducted in PCa patients with post-RP BCR and low PSA levels (≤2.0 ng/mL), in which the prevalence of distant localizations was 16%, and 8% of cases presented bone metastases. Fendler et al. [7] also showed a similar prevalence of bone metastases (~11%) in a subgroup of BCR patients with a PSA range of 0.5–1 ng/mL, comparable to the PSA IQR of our study. Although values of up to 18.7% were reported for M1b prevalence in similar cohorts [23], these findings are in line with the previous studies performed at our center on smaller sample sizes, in which 16.6–22.2% of cases showed distant recurrence (M1), while 10.3–12.5% presented bone localizations [11,24].

In regard to predictors of bone metastases at PSMA-PET in early BCR/BCP HSPC patients, limited evidence is currently available in literature. Recently, Bidakhvidi et al. [25] evaluated 175 ^18^F-PSMA-1007 PET scans performed in PCa patients with BCR after primary treatment. The PSA value at PET scan was proved to be a significant predictor of bone lesions (OR 1.007, *p* = 0.04), while both PSA value at scan time (IRR 1.003, *p* = 0.0002) and Gleason Score (IRR 1.57, *p* = 0.003) were independent predictors of the number of bone localizations. However, the reported PSA values were considerably higher compared to our study, both in the overall cohort (median: 1.6 ng/mL, range: 0.07–429 ng/mL), and in the subgroup treated with RP (median: 1.3 ng/mL, range: 0.07–250 ng/mL); moreover, patients with prior or ongoing treatment with ADT were also included, despite the potential influence of ADT on PSMA expression and the PSMA-PET detection rate. The association between PSA value at PET-scan and the presence/number of PSMA-avid bone metastasis was also documented by Pomykala et al. [26] in different clinical indications, including biochemical recurrence. However, as in the study by Bidakhvidi et al., the cohort PSA values were spread across a significantly wider range, with 22% having PSA values higher than 5 ng/mL. Both previously mentioned studies build upon prior literature evidence derived from other imaging techniques (such as bone scintigraphy [27]), in which the PSA absolute value and PSA kinetics were proved to be significant predictors of bone metastases in BCR HSPC patients after radical treatment (RP or RT). However, contrary to the aforementioned studies, our analysis was designed to include only patients with early BCR/BCP, in whom the tumor burden is lower and the salvage therapies are more effective. Therefore, the cohort PSA IQR was considerably narrower (0.38–1.04 ng/mL), and PSA kinetics measured with PSA doubling time resulted in being the more accurate predictor of bone metastatic involvement (OR: 0.93; 95% CI: 0.88–0.99; *p* = 0.026). This finding is also in line with previous evidence by Verburg et al. [28], in which PSAdt was the only significant independent determinant for bone metastases at ^68^Ga-PSMA-11 PET in a cohort of 155 recurrent PCa patients (*p* = 0.001).

In our study, the prevalence of bone metastases at PET imaging varied among different clinical settings, with lower rates for first-time BCR (5.6%), compared to BCR after SRT and BCP cases (14.4% and 13.0%, respectively). These results are in accordance with previous PSMA-PET studies, which documented different rates of overall positivity [11,23,24,29] and distant metastases (M1a/b/c) [23,24,29] among clinical settings, with first-time BCR representing the most favorable subgroup. At logistic regression analysis, our data showed clinical setting to be an independent predictor of skeletal involvement, with BCR patients previously treated with SRT on prostate-bed being at higher risk of bone metastases, compared to the first-time BCR patients (OR: 2.90; 95% CI: 1.32–6.35; *p* = 0.008). The higher incidence of bone metastases in post-SRT BCR patients is not surprising since these patients have already experienced a recurrence and have already failed a previous line of treatment. Regarding the BCP setting, only a marginal significance (*p* = 0.066) was found at univariate analysis. However, considering the high prevalence of bone metastases in this subgroup (13% vs. 14.4% in BCR after SRT cases), this result might be biased by the significantly lower sample size (*n* = 54 vs. 174 BCR after SRT vs. 215 first-time BCR). Moreover, it is possible that the T stage and PSAdt determined a confounding effect on the BCP setting, due to the higher prevalence of T stages ≥ 3a in this subgroup (66% vs. 51% in BCR after SRT vs. 54% in first-time BCR), and the lower PSAdt (3.4 months vs. 9.0 in BCR after SRT vs. 8.3 in first-time BCR). The hypothesis of a predictive role of BCP in bone recurrence is supported by previous studies by Ferdinandus J. et al. [30], Meijer et al. [31] and Farolfi et al. [32], in which high proportions (40%, 39%, and 33%, respectively) of BCP patients were found to already have distant metastatic localizations (≥miM1) at PSMA-PET. Moreover, in 2015 Bianchi et al. [33] already reported skeletal/visceral metastases to be the first site of recurrence in up to ~50% of BCP patients with a history of node-positive PCa treated with RP and extended pelvic lymph node dissection (ePLND).

Besides PSAdt and clinical setting, our study found the T stage ≥ 3a to be an independent predictor of bone metastases in BCR patients. This finding builds upon previous literature evidence in which the T stage, together with PSA and PSAdt [34,35,36], was proved to be a predictor of both overall positivity [11,24] and distant localizations at PSMA-PET in BCR patients [11].

Finally, contrary to the T stage, the ISUP grade did not reach significance at multivariate analysis, despite being previously shown to be a significant predictor of overall PSMA-PET positivity (with a possible stronger role in the BCP setting [29]).

### 4.1. Future Perspectives

The accuracy of the predictive model could be further improved by including additional promising parameters, such as the tumor PSMA-expression quantification and the alkaline phosphatase velocity (APV). Indeed, the ability of PSMA-PET to detect PCa localizations depends on the degree of PSMA expression by cancer cells, and Ferraro et al. [37] already showed how PSMA-PET can be falsely negative in 85% of BCR patients with a tumor PSMA-negativity ≥ 50%. Furthermore, increasing evidence has linked higher PSMA expression levels with more aggressive features (such as higher Gleason Scores, hormone resistance, and overall worse prognosis), which are also more likely to result in earlier metastatic spread.

Alkaline phosphatase velocity (APV) could also represent a promising predictive factor, since alkaline phosphatase is a known marker of bone-turnover, and rapid APV has shown potential in predicting distant metastasis-free survival in PCa patients with BCR after RP [38].

### 4.2. Limitations

This study is not exempt from limitations. First, due to ethical and practical reasons, positive PSMA-PET findings could not be histologically validated in all of the cases. However, images were independently evaluated by all of the clinicians in accordance with procedure Guidelines [19], and discrepancies were solved by consensus; moreover, high positive predictive values (PPV) for PSMA-PET were already established by previous studies [7,39]. Second, data commonly available in clinical practice did not include markers specifically linked to bone tropism: as previously stated, a better understanding of the interactions between prostate cancer cells and the bone microenvironment could allow an increase in the accuracy of the predictive model by including markers more closely involved in initial bone homing mechanisms. Finally, although a nomogram to predict PSMA-PET positivity was proposed [29,40], it could not be validated in our retrospective study since the nomogram was also originally built in a retrospective observational context, and, therefore, a prospective cohort would be needed for its validation. Moreover, such nomogram is not specific for the miM1b subgroup considered in this study, and a larger sample size would be required to perform nomogram validation.

## 5. Conclusions

The T stage (≥3a), clinical setting (BCR after SRT) and short PSAdt were proved to be significant predictors of bone metastases in early BCR/BCP HSPC patients, with a 7% risk increment for each single-unit decrement of PSAdt. These predictors could be used to further refine the indication for PSMA-PET in early BCR/BCP HSPC patients, thus leading to higher detection rates of bone disease and more personalized treatments.

## Figures and Tables

**Table 1:** Cohort characteristics (retrospective analysis of 443 ^68^Ga-PSMA-11 PET/CT scans).

Clinical Features		Median	IQR
Age (years)		74	68–78
iPSA (ng/mL)		7.85	5.73–12.00
PSA at PET scan (ng/mL)		0.60	0.38–1.04
PSAdt at PET scan (months)		8.2	4.2–14.6
PSAvel at PET scan (ng/mL/year)		0.5	0.3–1.2
**Clinical features**		**Frequency *n* (%)**	
ISUP Grade	1	49 (11.1%)	
	2	96 (21.7%)	
	3	137 (30.9%)	
	4	80 (18.1%)	
	5	57 (12.9%)	
	Missing	24 (5.4%)	
pT stage	<3a	192 (43.3%)	
	≥3a	228 (51.5%)	
	Missing	23 (5.2%)	
pN stage	N1	42 (9.5%)	
R (margin)	R1	160 (36.1%)	
Time to PSA relapse (months)	>12	314 (70.9%)	
	≤12	124 (28.0%)	
Primary therapy	RP ± LND ± adjuvant RT	417 (94.1%)	
	Primary RT	20 (4.5%)	
Clinical stage of PSA failure at PSMA PET/CT	First-time BCR (subgroup-1)	215 (48.5%)	
	PSA relapse after prostate-bed SRT (subgroup-2)	174 (39.3%)	
	BCP after RP (subgroup-3)	54 (12.2%)	

**Table 2:** Univariate and multivariate logistic regression models for bone disease at PSMA-PET.

Potential Predictors of Bone Recurrence	Univariate Model			Multivariate Model		
	OR	95% CI	*p*	OR	95% CI	*p*
T stage (≥3a)	2.59	1.26–5.30	0.009	2.52	1.13–5.60	0.024
ISUP Grade (≥4)	2.37	1.24–4.54	0.009	-	-	0.146
Clinical Setting BCR after SRT vs. first-BCR BCP vs. first-BCR						
	2.84	1.38–5.83	0.005	2.90	1.32–6.35	0.008
	2.52	0.94–6.74	0.066	-	-	-
PSA (ng/mL) at PSMA-PET	0.98	0.82–1.17	0.84	-	-	-
Time to recurrence (months)	1.00	0.99–1.01	0.46	-	-	-
PSA doubling time (months)	0.91	0.86–0.97	0.004	0.93	0.88–0.99	0.026
PSA velocity (ng/mL/year)	1.03	0.98–1.09	0.27	-	-	-

## Data Availability

The data are not publicly available due to privacy constraints.
